# Evaluation of Bait Acceptance and Immune Response in Local Dogs during an Oral Rabies Vaccination Field Study in Morocco

**DOI:** 10.3390/tropicalmed9070142

**Published:** 2024-06-26

**Authors:** Nadia Aboulfidaa, Florence Cliquet, Emmanuelle Robardet, Sami Darkaoui, Marine Wasniewski, Christian Kaiser, Katharina Bobe, Ad Vos, Ouafaa Fassi Fihri

**Affiliations:** 1 Division of Pharmacy and Veterinary Inputs, National Food Safety Office, Rabat BP 4509, Morocco; nad_ab@hotmail.fr (N.A.); sami.darkaoui@onssa.gov.ma (S.D.); 2 ANSES—Nancy Laboratory for Rabies and Wildlife, French Agency for Food, Environmental and Occupational Health & Safety, European Union Reference Laboratory for Rabies, WHO Collaborating Centre for Research and Management in Zoonoses Control, WOAH Reference Laboratory for Rabies, Technopôle Agricole et Vétérinaire de Pixérécourt, 54220 Malzéville, France; florence.cliquet@anses.fr (F.C.); emmanuelle.robardet@anses.fr (E.R.); marine.wasniewski@anses.fr (M.W.); 3 TEW Servicegesellschaft GmbH, Am Pharmapark (formerly at Ceva Innovation Center), 06861 Dessau-Rosslau, Germany; christian.kaiser@tew-service.de; 4 Ceva Innovation Center GmbH, Am Pharmapark, 06861 Dessau-Rosslau, Germany; katharina.bobe@ceva.com; 5 Department of Pathology and Veterinary Public Health, Agronomic and Veterinary Institute Hassan II, Rabat BP 6202, Morocco; o.fassifihri@iav.ac.ma

**Keywords:** bait, SPBN GASGAS, oral vaccination, immunogenicity, dogs, Morocco, rabies

## Abstract

The objective of this study was to evaluate the bait preference of three selected bait types by local dogs and the induced immunogenicity of the oral rabies vaccine strain SPBN GASGAS in Morocco. The vaccine strain, combined with different bait types, has been tested in many different settings, but not yet in northern Africa. Overall, bait consumption and preference were similar in other studies using the same materials (bait type and sachet). The intestine bait had the highest acceptance rate (97.6%, 95%CI: 87.4–99.9), followed by the egg bait (83.0%, 95%CI: 69.2–92.4). Only 52% (95%CI: 37.4–66.3) of the dogs showed an interest in the fish meal bait. However, considering the successful release of the contents of the sachet (blue-dyed water) into the oral cavity, the egg bait (65.7%, 95%CI: 47.8–80.9) scored better than the intestine bait (51.7%, 95%CI: 32.5–70.6). The dogs selected for the immunogenicity study were offered the egg bait containing a sachet filled with SPBN GASGAS (3.0 mL, 10^7.5^ FFU/mL) or were given the same dose by direct oral administration (d.o.a.). In addition, several dogs were vaccinated by the parenteral route (s.c.) using a commercially available inactivated rabies vaccine. Unfortunately, due to the COVID-19 pandemic and subsequent travel restrictions, it was not possible to collect blood samples directly after vaccination. The blood samples were collected pre-vaccination and on five occasions between 450 and 1088 days post vaccination. The seroconversion rate, as determined for rabies-virus-neutralizing antibodies by the FAVN test, was significantly lower than that found for binding antibodies, as determined by ELISA, for all blood samples collected post vaccination. No treatment effect (bait, d.o.a., s.c.) could be seen in the seroconversion rate. At 15 months post vaccination, 84.2% of the dogs offered vaccine bait still tested sero-positive in ELISA. Only after 3 years was a clear drop in the seroconversion rate observed in all three treatment groups. This study confirms the long-term immunogenicity of the oral rabies vaccine SPBN GASGAS in dogs under field conditions.

## 1. Introduction

In Morocco, rabies is still endemic throughout the country, with dogs being the main reservoir species [1]. The spread of this disease can be avoided by preventing virus transmission at the source by vaccinating a sufficient proportion of the dog population. However, the estimated vaccination coverage of the dog population in Morocco is low, especially in rural areas [2,3]. The reasons for this low coverage are manifold, for example, among others, a large proportion of free-roaming dogs are not accessible for parenteral vaccination. Oral rabies vaccination (ORV) of these dogs has been proposed as a complementary tool to increase the overall vaccination coverage—especially in the free-roaming dog population, which plays a key role in rabies transmission—with the ultimate goal of the elimination of dog-mediated rabies [4,5,6]. Rabies elimination from the dog population in Morocco would not only benefit the country itself, but also neighboring countries in the region and the European Union. For example, 14 (66.7%) of the 21 rabies cases attributed to pets from rabies-endemic countries reported in western Europe during 2001–2013 originated from Morocco [7,8,9,10]. Previous studies have investigated the feasibility of ORV in Morocco [11]. The objective of the present study was to confirm the acceptance of a suitable bait candidate and, subsequently, to test the immunogenicity of the oral live rabies virus vaccine (SPBN GASGAS) in local dogs. The selected egg-flavored bait and vaccine virus have been tested extensively in other regions and experimental settings [12,13,14,15,16,17,18,19,20]; however, so far, data from northern Africa are lacking.

## 2. Materials and Methods

### 2.1. Study Area

**Figure 1:**
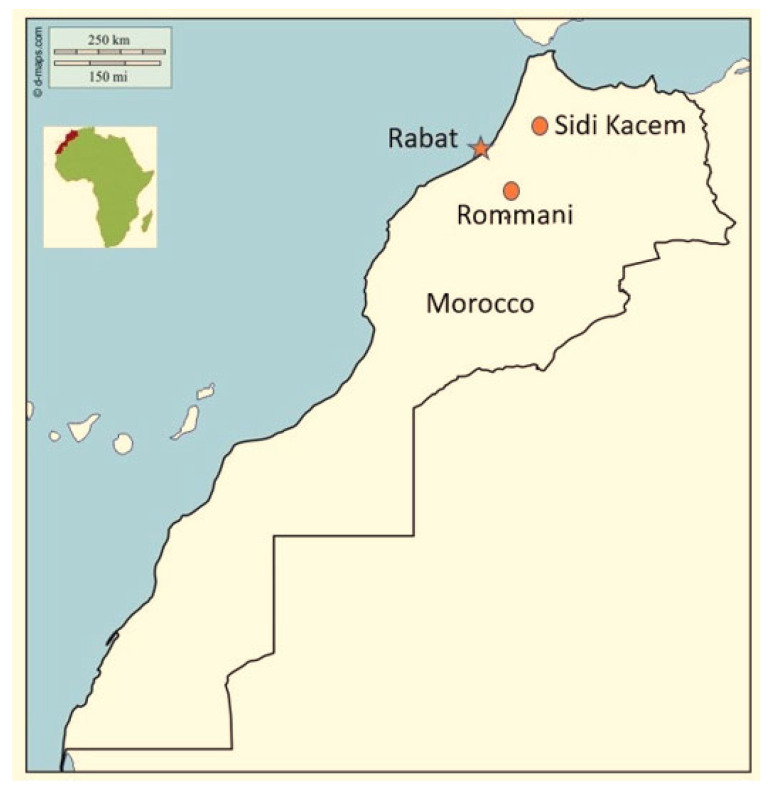
Bait acceptance and serology investigations were conducted, near the capital, Rabat (orange star icon), in the rural areas surrounding the cities (orange circle icons) of Sidi Kacem and Rommani in Morocco, respectively.

This bait acceptance study was performed in a rural area near Sidi Kacem City in the region of Rabat-Salé-Zemmour-Zaër, located 137 km northeast of Rabat, the capital of Morocco, with an area of 4060 km^2^ and a human population of 90,000. Rabies is highly endemic in this region [1]. The immunogenicity study area was situated in a rural area near the city of Rommani, in the region of Rabat-Salé-Zemmour-Zaër, located 78 km south of Rabat, with an area of 9580 km^2^ and a human population of 3,123,595 (Figure 1).

### 2.2. Bait Acceptance Study

This study was conducted in the area surrounding Sidi Kacem, and the same bait types and study protocol as seen in previous studies were used [12,13,14]. Briefly, two manufactured baits, fish (meal)- and egg (flavored)-bait, and a bait made from locally available boiled bovine intestine segments, were used. The bait type offered to each individual dog was pre-determined at random using specialized software (www.randomizer.org, accessed on 17 February 2020). The sachet incorporated into the different baits contained 3.0 mL of blue-dyed water. The baits were stored in a freezer at −20 °C prior to the field study and kept in cool boxes during the actual field studies. During a systematic coverage of the area, every dog encountered was offered a single bait. If the dog had an owner that could be identified, oral consent was obtained before the bait was offered to the dog. The observations were recorded on a special form for each individual dog, including detailed information on bait handling, such as interested or not and bait consumption (amount and duration). Furthermore, if possible, it was reported if the sachet was perforated or not and if it was discarded or swallowed. Finally, based on these observations, including coloring of the oral cavity due to the release of the sachet contents, an evaluation of the vaccination attempt was made (successfully vaccinated or not). Additionally, general information on the dog was collected, including if the dog was owned or not, free-roaming or restricted, alone or with other dogs, age category, sex, and size. 

### 2.3. Immunogenicity Study

Regarding the immunogenicity study, a similar protocol as that seen in previous studies was used [17,18,19]. Forty-six (46) dogs aged > 3 months were selected after the owners’ consent was obtained. All dogs were clinically healthy, and the animals were individually marked with a transponder chip (BackHome BIOTEC Tronspondeur, Virbac Tierarzneimittel, GmbH Bad Oldesloe—Germany) for identification purposes. The dogs included in this study should not have been vaccinated previously against rabies. Hence, a blood sample (B0) was collected 14 days prior to vaccination confirming the absence of antibodies against rabies. The owners were informed of the visit of the study team several days prior to vaccination and blood sampling. The study animals were fed by their owners as usual, and no special diet was provided to the dogs during this study.

The animals were divided into three treatment groups. The largest group (n = 25) was offered an egg bait containing a sachet filled with 3.0 mL of SPBN GASGAS (10^7.5^ FFU/mL). If the dogs would not accept the bait, the animals (n = 10) were offered the same volume and dose of SPBN GASGAS by direct oral administration (d.o.a.), or the dogs (n = 10) were vaccinated by the parenteral route (subcutaneously (s.c.)) using 1.0 mL of Rabisin Multi (BIAH, Lyon—France) as a positive control group. The selected oral rabies vaccine strain, SPBN GASGAS, is a genetically engineered 3rd generation oral rabies virus vaccine that is highly attenuated through site-directed mutations and licensed for oral vaccination of red foxes and raccoon dogs in the EU [21,22].

Initially, it was scheduled that blood samples of the dogs would be collected starting 28 days post vaccination (dpv). Unfortunately, (inter)national travel restrictions put in place due to the emergence of the COVID-19 pandemic prevented us from visiting the study area for blood sampling. Consequently, the first blood samples post vaccination (B1) could only be collected 450 dpv. Additional blood samples from the dogs were acquired on 643 (B2), 723 (B3), 788 (B4), and 1088 dpv (B5). 

The blood samples were collected from the large superficial veins on the front of a foreleg or on the outside of a hind leg (e.g., V. cephalica antebrachii, V. saphena), into 5-mL uncoated blood collection tubes (S-Monovette, Sarstedt, Nümbrecht, Germany) and centrifuged at 3500× *g* for 15 min. Subsequently, the sera were stored at −20 °C until laboratory analysis for the presence of virus antibodies.

### 2.4. Serological Tests

The serum samples were analyzed for the presence of rabies-virus-binding antibodies (rVBA) using a commercial blocking enzyme-linked immunosorbent assay kit (BioPro Rabies ELISA, O.K. Servis BioPro, Prague, Czech Republic) following the instructions of the manufacturer essentially as described [23]. Briefly, the ELISA kit is an indirect blocking test that detects rabies virus antibodies. The wells of microplates are coated with rabies antigen. Diluted samples (including positive and negative controls) are incubated into the wells and, after washing, biotinylated anti-rabies antibody is added to the wells. After washing, Streptavidin peroxidase conjugate is added. After another washing step, a Tetramethylbenzidine (TMB) substrate solution is added to the wells, forming a blue compound and becoming yellow after stopping the reaction. The intensity of the color is read at 450 nm and the decrease in the intensity compared with the negative control is proportional to the amount of blocking antibodies in the investigated sample. The percentage of blocking (PB) was calculated for each sample according to the manufacturer’s specifications.

Furthermore, the fluorescent antibody virus neutralization test (FAVN test) was used for the detection of rabies-virus-neutralizing antibodies (rVNA) [24]. Prior to testing, the sera were inactivated for 30 min at 56 °C. Briefly, in a 96-well cell culture microplate, 50 µL of rabies virus (strain CVS-11, ATCC number VR 959) solution, diluted to 100 TCID50/mL with D-MEM containing 10% FCS, was added to 50 µL of each threefold dilution of serum and incubated for 1 h in 5% CO_2_ at 37 °C. Then, 50 µL of a BHK 21-cell (BHK-21 C13, ATCC number CCL-10) suspension at 4 × 10^5^ cells/mL in D-MEM containing 10% FCS was added to all tested wells and incubated for 48 h in 5% CO_2_ at 37 °C. After incubation, all test wells were fixed and stained using an anti-rabies virus fluorescein isothiocyanate (FITC)-conjugated monoclonal antibody (Fujirebio Diagnostics). The titer expressed in international units per ml (IU/mL) of each serum sample was calculated by comparison with the WOAH anti-rabies-positive reference serum [25]. 

For the ELISA and FAVN tests, cut-offs for sero-positivity of ≥40% PB and ≥0.24 IU/mL (international units per milliliter) were used, respectively. 

### 2.5. Statistical Assays

Statistical analyses were carried out using GraphPad Prism 7 (GraphPad Software Inc., San Diego, CA, USA).

## 3. Results

### 3.1. Bait Acceptance

A total of 184 dogs were offered bait, but the forms of only 139 attempts were suitable for data analysis. Several forms contained information that invalidated the observation. For example, sometimes essential data like the bait type were not recorded. Finally, forms containing contradicting data were removed as well (for example, when the bait offered was recorded as ‘ignored’ but subsequently the sachet was ‘perforated’ and ‘discarded’).

**Figure 2:**
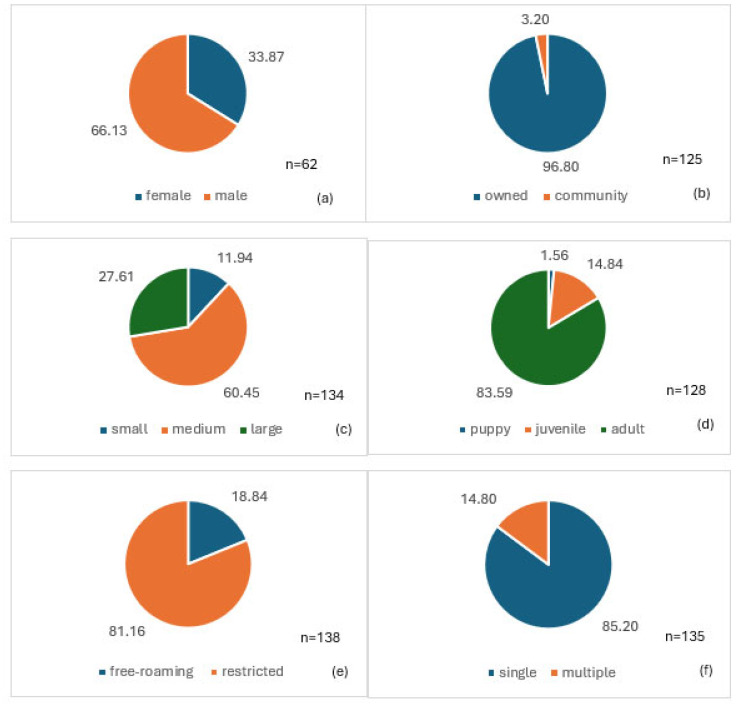
General description of the dogs offered bait during the acceptance study: (**a**) sex (male or female), (**b**) ownership (owned or community), (**c**) size of dog (small, medium, or large), (**d**) age (puppy ≤ 3 months, juvenile = 3–12 months, adult ≥ 12 months), (**e**) level of confinement (restricted (e.g., garden) or free-roaming), and (**f**) if the dog was alone (single) or together with other dogs (multiple) when offered the bait.

A total of 50 dogs were offered fish bait, 42 dogs were offered intestine bait, and 47 dogs were offered egg bait. The general information of the dogs included in the analysis is summarized in Figure 2. Most of the dogs offered bait were medium-sized adult male owned dogs that were single and restricted. 

Overall, 76.3% of the dogs showed interest in the baits offered (Table 1). However, there were clear differences observed between the bait types (Chi^2^ = 28.0, df = 2, *p* < 0.0001). Only one dog showed no interest in the intestine bait, meanwhile 83% of the dogs showed an interest in the egg bait and only 52% of the dogs were interested in the fish bait. The intestine bait was consumed more often (100%) than the egg bait (94.9%), and the fish bait (88.5%) was the least preferred by the dogs that showed interest. However, the estimated vaccination success was the highest for the egg bait when consumed (65.7%) and also when corrected for the animals that were offered the bait but were not interested (51.1%). The fish bait obtained a very low vaccination coverage.

The relatively high vaccination success rate of the egg bait can be explained by the fact that the sachet in this bait was more often perforated than the sachets in the other two bait types (Table 2). The highly attractive intestine bait was most often completely consumed within a very short time (<10 s) and without much chewing, resulting in swallowing the sachet without it being perforated and, consequently, no release of the contents in the oral cavity.

### 3.2. Immunogenicity Study

Initially, 45 dogs were included in this study. All of the dogs, except one, tested sero-negative in ELISA (<40%PB) prior to vaccination. The sero-positive animal was still vaccinated by the parenteral route but was excluded from analysis. Another extremely aggressive dog refused the bait and was also excluded from this study. Hence, a total of 43 dogs were available for post vaccination antibody screening. However, the dogs could not always be located during the subsequent sampling time points. For example, if the owner (and dog) had moved, the dog or owner were not at home during the visit, or if dog had died. Hence, the number of samples collected and subsequently tested varied and decreased with time (Table 3). Four B2-samples taken from dogs offered bait were only tested in ELISA and not with the FAVN test.

**Figure 3:**
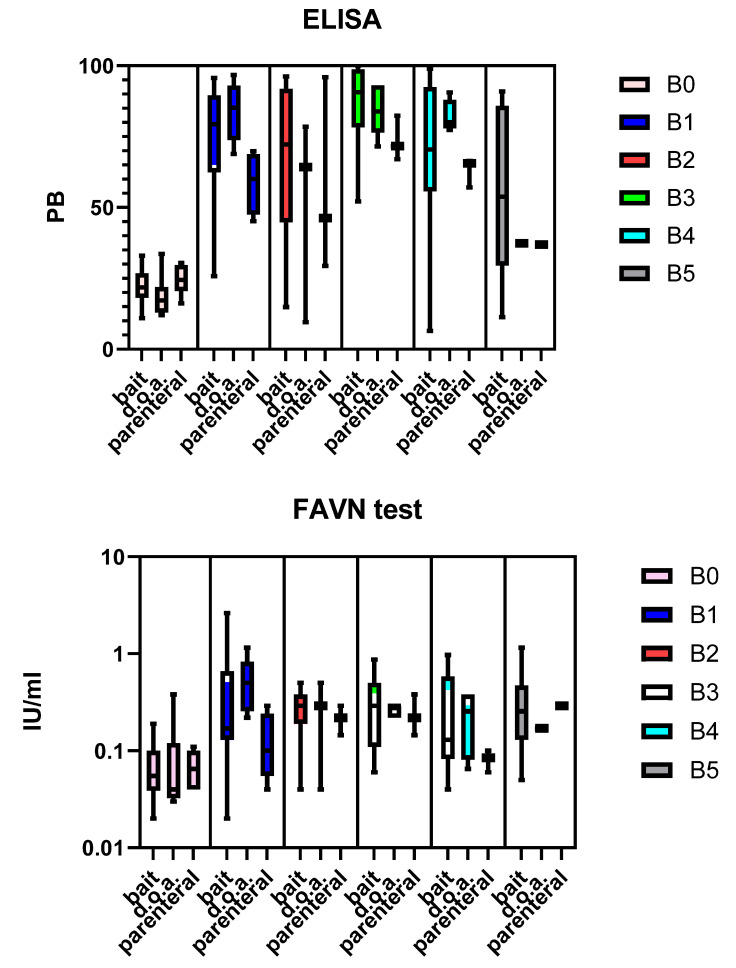
Box plot showing the levels of rVBA (ELISA) and rVNA (FAVN test) of the blood samples collected, grouped per treatment and sampling point.

The levels of rVBA and rVNA of the different blood samples for the dogs offered bait are shown in Figure 3. The mean levels of rVBA and rVNA and seroconversion rate per treatment group and sampling point are summarized in Table 4. The values of the individual animals can be found in Appendix A. The seroconversion rates, as determined for rVNA, were almost always lower than those found for rVBA for all collected blood samples post vaccination.

Four animals that tested sero-negative in ELISA after vaccination (B1 and/or B2) tested sero-positive during the subsequent sampling point(s). Three years post vaccination, 50% (4/8) of the dogs still tested positive for rVBA in ELISA. Most of the dogs tested sero-negative for rVNA in the FAVN test.

## 4. Discussion

The bait acceptance rate was comparable with other studies using the same bait types and sachets [12,13,14]. However, the estimated vaccination success rate found during this bait acceptance study was relatively low compared to that of other studies, especially for the fish bait. Only 27.3% of the dogs that consumed the fish bait were considered ‘vaccinated.’ For the other two bait types—the intestine (51.7%) and egg bait (65.7%)—the vaccination rate was much higher but still lower than that observed in Navajo Nation, Thailand, and Indonesia (80–95%) [12,13,14]. However, the general conclusions correspond with those obtained during the other studies, i.e., the egg bait had the highest success rate, although the intestine bait was more often accepted. This is a result of the relatively high effectiveness of the egg bait in releasing the content of the sachet in the oral cavity in contrast to the intestine bait. The latter is often swallowed without much chewing and, thus, without perforation of the sachet. Consequently, the content of the sachet is not released into the oral cavity. The reasons for the relatively low bait acceptance rate are most likely a result of multiple factors. Often, the dogs were brought to a central location where many people and other dogs were gathered. This could have induced stress and affected bait acceptance negatively, as was observed in Namibia [18]. In the other studies, baits were offered to the dogs at the location where the dogs were encountered and, thus, at familiar sites [12,13,14]. Furthermore, ORV is targeted at dogs that are not accessible for parenteral vaccination and, in this study, most of the dogs were owned and restricted. Thus, most of the animals did not represent the target dog population for ORV, i.e., free-roaming dogs. However, no difference in bait acceptance was observed between the restricted and free-roaming dogs in Navajo Nation and Bali—Indonesia [12,14]. 

The results of the immunogenicity study are more difficult to compare with other studies using the same vaccine construct, as the latter included only blood samples collected shortly after vaccination [17,18,19,20]. Due to the travel restrictions put in place during the COVID-19 pandemic, the first blood samples could only be collected approximately 15 months post vaccination during the present study. With the oral rabies vaccine SPBN GASGAS, only one other long-term immunity study in Thai shelter dogs has been conducted. Here, similar seroconversion rates found using ELISA were observed 30 months post vaccination. More than 80% of the dogs offered a single vaccine bait tested sero-positive (ELISA) up to 26 months post vaccination [16]. In Morocco, the seroconversion rate based on rVBA dropped only at the last sampling point (36 months post vaccination). Unfortunately, the sample size decreased considerably during this study and, thus, the estimated 95% confidence interval of the seroconversion rate is rather large.

Several dogs initially tested sero-negative post vaccination (B1 and B2) but, during subsequent sampling points, the animals were sero-positive. The two possible explanations are (1) the animals received a booster vaccination or (2) naturally acquired immunity after a non-lethal exposure to the rabies virus. A booster vaccination is most unlikely, as the practicing regional veterinarian received a list of the dogs with the identification number with the request to exclude these animals from rabies vaccination. In addition, the veterinarian declared that, due to the pandemic, he did not receive any rabies vaccines during the study period, including B0 to B4. As the study area is located in a region where dog rabies is endemic, it cannot be excluded that these dogs came into contact with rabid dogs. A study in Tanzania showed that a proportion of healthy unvaccinated dogs had detectable levels of rabies antibodies [26]. Here, a number of dogs bitten by two rabies-suspected dogs survived exposure, of which several subsequently seroconverted [27]. 

The results of this study underscore previous immunogenicity studies with SPBN GASGAS in dogs, in that the seroconversion rate based on rVNA is relatively low in contrast to rVBA using the commercially available BioPro ELISA kit [15,16,17,18,19,20]. It has been shown that the cut-off for sero-positivity used in this ELISA is a much better surrogate for protective immunity than another virus-neutralizing antibody assay, RFFIT [15,28]. In addition, several dogs vaccinated with the 2nd generation oral rabies virus vaccine SAG2 survived a challenge infection without detectable levels of rVNA [29]. In this study, the cut-off threshold of sero-positivity in virus-neutralizing antibody assays was lowered from the commonly used 0.5 to 0.24 IU/mL. The 0.5 IU/mL threshold is often misused; moreover, it was not established as a minimum level for protective immunity but initially solely as an indication of adequate vaccination for humans [30]. Subsequently, this threshold is also applied for animals, including dogs, and is also used as proof of adequate antibody titer for international movements of animals [31]. However, it has been shown that a lower threshold (0.24 IU/mL) can be used for dogs as a surrogate for protective immunity [32,33].

## 5. Conclusions

It can be concluded that the consumption of egg bait containing a sachet filled with the oral rabies virus vaccine, SPBN GASGAS, induced long-term immunity, as shown by the presence of rVBA in local dogs in Morocco.

## Figures and Tables

**Table 1:** Bait acceptance and subsequent ‘vaccination rate’ determined by direct observation of bait consumption by the dog (interested—percentage of dogs offered bait that had direct contact with bait (licking, sniffing), consumed (if interested)—proportion of dogs that showed interest and subsequently consumed the bait offered partially or completely, vaccinated (if consumed)—percentage of dogs that consumed the bait and were considered vaccinated, vaccinated (offered)—percentage of dogs that were offered bait and were subsequently considered vaccinated, irrespective of if the dogs showed interest and/or consumed the bait). The 95% confidence interval is shown in brackets. Notice: Sometimes the outcome (vaccination assessment) was unknown. Hence, the sample size between rows does not always match.

	Interested	Consumed (If Interested)	Vaccinated * (If Consumed)	Vaccinated * (Offered)
Bait type	% (n/N)	% (n/N)	% (n/N)	% (n/N)
Egg	83.0 (39/47) [69.2–92.4]	94.9 (37/39) [82.7–99.4]	65.7 (23/35) [47.8–80.9]	51.1 (23/45) [35.8–66.3]
Fish	52.0 (26/50) [37.4–66.3]	88.5 (23/26) [69.8–97.6]	27.3 (6/22) [10.7–50.2]	12.5 (6/48) [4.7–25.2]
Intestine	97.6 (41/42) [87.4–99.9]	100.0 (41/41) [91.4–100]	51.7 (15//29) [32.5–70.6]	50.0 (15/30) [31.3–68.7]
Total	76.3 (106/139) [68.3–83.1]	95.3 (101/106) [95.3–89.3]	51.2 (44/86) [40.1–62.1]	33.1 (44/133) [25.2–41.8]

* A dog was considered ‘vaccinated’ when a clear blue staining of the oral cavity, incl. the tongue, was visible and/or the discarded perforated sachet was recollected.

**Table 2:** Number and percentage of sachets discarded or not (swallowed) and perforated or not. For the calculation of the percentages, the number of ‘unknowns’ has been excluded. The 95% confidence interval is shown in brackets.

Bait Type	Discarded			Perforated		
	Unknown	Yes	No	Unknown	Yes	No
Egg	12	20 (80.0%) [62.5–91.8]	5 (20.0%) [8.2–37.5]	4	26 (78.8%) [63.8–89.6]	7 (21.2%) [10.4–36.2]
Fish	11	11 (91.7%) [66.1–99.6]	1 (8.3%) [0.4–33.9]	1	9 (40.9%) [23.3–60.5]	13 (59.1%) [39.5–76.7]
Intestine	5	5 (13.9%) [5.6–27.0]	31 (86.1%) [73.0–94.4]	19	14 (63.6%) [43.9–80.4]	8 (36.4%) [19.6–56.1]
Total	28	36 (49.3) [39.2–59.5]	37 (50.7%) [40.5–60.8]	24	49 (63.6%) [53.7–72.8]	28 (36.4%) [27.2–46.3]

**Table 3:** Number of samples taken from the dogs on the different post-vaccination visits for the different treatment groups (B—Blood samples collected at different times after vaccination; dpv—days post vaccination; d.o.a.—direct oral instillation).

Sample	B1	B2	B3	B4	B5
Date	27.05.21	09.12.21	01.03.22	13.05.22	14.03.23
dpv	450	643	723	788	1088
Number of dogs not sampled	15	22	23	23	33
-not found *	15	12	10	8	9
-died	0	9	12	14	24
-aggressive	0	1	1	1	0
Number of dogs sampled	28	21	20	20	10
-bait	19	15	12	13	8
-d.o.a.	5	3	5	4	1
-parenteral	4	3	3	3	1

* Not found: owner/dog moved or not present during visit.

**Table 4:** Mean rabies-virus-binding antibodies—ELISA and neutralizing antibody (FAVN test) response in serum samples collected from the dogs for the three treatment groups and the subsequent seroconversion rate. Only the dogs for which at least one sample post vaccination was available were included. Sometimes, for the FAVN test results, only an upper limit (e.g., ≤0.30 IU/mL) was available and no exact value. In this case, half of this upper limit value was used for the statistical analysis (s.d.—standard deviation, n—number of sero-positive samples, N—number of samples, 95%CI—95% confidence interval).

Blood Sample	B0		B1		B2		B3		B4		B5	
Assay	ELISA	FAVN	ELISA	FAVN	ELISA	FAVN	ELISA	FAVN	ELISA	FAVN	ELISA	FAVN
Bait												
Mean	23.0	0.07	72.6	0.48	65.9	0.28	86.0	0.33	70.4	0.31	54.7	0.36
N	22	21	19	19	15	11	12	12	13	13	8	8
s.d.	5.6	0.05	21.6	0.62	26.4	0.15	17.3	0.26	25.3	0.30	30.7	0.35
Seroconversion												
n/N	0/22	0/21	16/19	9/19	12/15	6/11	12/12	7/12	12/13	5/13	4/8	4/8
rate (%)	0	0	84.2	47.4	80.0	54.5	100	58.3	92.3	38.5	50.0	50.0
lower 95%CI	0	0	64.1	27.4	56.0	27.1	77.9	31.5	68.4	16.6	19.3	19.3
upper 95%CI	12.7	13.3	95.6	68.0	94.3	80.0	100	81.9	99.6	64.5	80.7	80.7
d.o.a.												
mean	17.5	0.09	83.7	0.53	50.7	0.28	84.6	0.26	82.0	0.24	37.4	0.17
N	7	7	5	5	3	3	5	5	4	4	1	1
s.d.	63.9	0.13	10.6	0.37	36.4	0.23	9.0	0.04	5.8	0.17	-	-
Seroconversion												
n/N	0/7	1/7	5/5	4/5	2/3	2/3	5/5	3/5	4/4	2/4	0/1	0/1
rate (%)	0	14.3	100	80.0	66.7	66.7	100	60.0	100	50.0	0	0
lower 95%CI	0	0.7	54.9	34.3	13.5	13.5	54.9	18.9	47.3	9.8	0	0
upper 95%CI	34.8	52.1	100	99.0	98.3	98.3	100	92.4	100	90.2	95	95
Parenteral												
mean	24.8	0.07	58.7	0.13	57.2	0.22	73.7	0.25	63.0	0.08	36.9	0.29
N	4	4	4	4	3	3	3	3	3	3	1	1
s.d.	3.7	0.03	11.2	0.11	34.6	0.07	7.9	0.12	5.2	0.02	-	-
Seroconversion												
n/N	0/4	0/4	4/4	1/4	2/3	1/3	3/3	1/3	3/3	0/3	0/1	1/1
rate (%)	0	0	100	25.0	66.7	33.3	100	33.3	100	0	0	100
lower 95%CI	0	0	47.3	1.3	13.5	1.7	36.8	1.7	36.8	0	0	5.0
upper 95%CI	52.7	52.7	100	75.1	98.3	86.5	100	86.5	100	63.2	95.0	100

## Data Availability

The original data can be provided upon reasonable request and should be directed to the corresponding author.

## Appendix A

Individual rabies-virus-binding—(ELISA—percentage blocking (PB)) and neutralizing antibody (FAVN test—IU/mL) response in serum samples collected from the dogs per treatment group (dpv—days post vaccination—not tested). Sometimes, for the FAVN results, only an upper limit (e.g., ≤0.30 IU/mL) was available and no exact value. In this case, half of this upper limit value was used for statistical analysis (GMT—geometric mean titer; SD—standard deviation). Only dogs sampled at least once post vaccination have been included, and the sero-positive dog in ELISA prior to vaccination has been excluded.

**Table A1:** Dogs offered vaccine bait.

	B0		B1		B2		B3		B4		B5	
	−14 dpv		450 dpv		643 dpv		723 dpv		788 dpv		1088 dpv	
Nr	ELISA	FAVN	ELISA	FAVN	ELISA	FAVN	ELISA	FAVN	ELISA	FAVN	ELISA	FAVN
1	31.65	0.1	-	-	14.85	≤0.38	-	-	-	-	-	-
2	21.82	0.07	80.91	≤0.29	44.7	-	76.65	≤0.22	77.98	≤0.22	-	-
3	17.74	0.13	74.9	0.07	47.31	≤0.38	52.13	0.06	58.32	0.1	28.1	0.13
4	18.93	≤0.07	-	-	-	-	-	-	-	-	-	-
5	25.89	≤0.17	79.36	0.38	22.19	-	101.26	0.87	95.38	0.87	-	-
6	19.33	-	-	-	72.19	-	87.79	0.29	-	-	-	-
7	32.93	0.04	37.66	0.17	39.72	0.22	-	-	66.39	0.04	-	-
8	20.08	≤0.06	90.46	≤0.1	-	-	-	-	-	-	-	-
9	26.82	0.04	55.5	0.66	69.41	0.04	-	-	53.11	0.1	11.3	0.13
10	14.51	≤0.07	65.77	0.04	-	-	-	-	-	-	-	-
11	14.82	≤0.1	79.25	≤0.29	-	-	-	-	-	-	-	-
12	21.95	0.17	30.39	0.17	95.19	0.38	83.14	≤0.29	6.47	0.66	33.5	0.22
13	25.27	0.07	86.93	0.13	76.1	0.1	84.4	≤0.22	49.92	0.06	-	-
14	31.12	≤0.38	67.63	0.38	67.86	0.5	97.38	0.66	67.48	≤0.13	36.7	≤0.1
15	20.62	≤0.06	62.34	≤0.1	-	-	-	-	-	-	-	-
16	30.85	0.04	25.83	0.66	96.17	0.38	54.84	0.38	93.78	0.38	88.6	0.38
17	28.99	0.07	90.77	1.15	91.84	0.5	104.65	0.5	98.99	0.66	90.9	1.15
18	23.15	0.04	86.1	2.62	-	-	-	-	-	-	-	-
19	24.83	0.06	85.06	0.17	86.95	0.29	99.13	0.29	91.09	0.13	77.6	0.29
20	20.40	≤0.06	95.64	0.66	72.43	0.29	93.51	≤0.13	85.21	0.22	-	-
21	16.37	≤0.13	95.54	0.87	-	-		-	-	-	-	-
22	18.10	0.1	89.52	0.87	91.92	-	96.9	0.5	70.42	0.5	70.09	0.5

**Table A2:** Dogs receiving the oral rabies vaccine by direct oral administration (d.o.a.).

	B0		B1		B2		B3		B4		B5	
	−14 dpv		450 dpv		643 dpv		723 dpv		788 dpv		1088 dpv	
Nr	ELISA	FAVN	ELISA	FAVN	ELISA	FAVN	ELISA	FAVN	ELISA	FAVN	ELISA	FAVN
1	20.53	≤0.06	85.17	0.29	-	-	83.91	0.22	79.66	0.13	-	-
2	12.73	≤0.06	89.21	0.5	-	-	-		-	-	-	-
3	23.32	0.38	78.63	0.5	78.47	0.5	81.3	0.29	77.31	0.38	-	-
4	17.17	0.07	96.68	1.15	-	-	93.02	0.29	-	-	-	-
5	13.18	0.04	-	-	9.54	0.04	93.02	0.29	90.5	0.38	-	-
6	15.97	≤0.13	-	-	-	-	-	-	-	-	-	-
7	19.82	≤0.07	68.88	0.22	64.19	0.29	71.51	0.22	80.34	≤0.13	37.4	0.17

**Table A3:** Dogs vaccinated by the parenteral route using an inactivated rabies vaccine.

	B0		B1		B2		B3		B4		B5	
	−14 dpv		450 dpv		643 dpv		723 dpv		788 dpv		1088 dpv	
Nr	ELISA	FAVN	ELISA	FAVN	ELISA	FAVN	ELISA	FAVN	ELISA	FAVN	ELISA	FAVN
1	29.7	0.07	45.12	0.1	29.36	0.22	82.36	0.22	66.39	0.06	-	-
2	20.58	≤0.22	65.66	0.29	46.25	≤0.29	67.05	≤0.29	57.06	≤0.17	-	-
3	24.43	0.04	69.81	0.1	95.92	0.29	71.61	0.38	63.53	0.1	36.9	0.29
4	24.52	0.04	54.36	0.04	-	-	-	-	-	-	-	-
